# The Sense of Agency Scale: A Measure of Consciously Perceived Control over One's Mind, Body, and the Immediate Environment

**DOI:** 10.3389/fpsyg.2017.01552

**Published:** 2017-09-12

**Authors:** Adam Tapal, Ela Oren, Reuven Dar, Baruch Eitam

**Affiliations:** ^1^Department of Psychology, Faculty of Social Studies, Masaryk University Brno, Czechia; ^2^The School of Psychological Sciences, Tel Aviv University Tel Aviv-Yafo, Israel; ^3^Motivated Cognition Lab, Department of Psychology, University of Haifa Haifa, Israel

**Keywords:** sense of agency, agency-related beliefs, direct measures of agency, expectancy, judgment of agency

## Abstract

The sense of agency (SoA) is defined as “the registration that I am the initiator of my actions.” Both “direct” and “indirect” measurement of SoA has focused on specific contextualized perceptual events, however it has also been demonstrated that “higher level” cognitions seemingly affect the SoA. We designed a measure of person's general, context-free beliefs about having core agency—the Sense of Agency Scale (SoAS). An exploratory (EFA) and confirmatory (CFA) factor analyses on samples of 236 (Study 1) and 408 (Study 2) participants yielded two correlated factors we labeled Sense of Positive Agency (SoPA) and Sense of Negative Agency (SoNA). The construct validity of SoAS is demonstrated by its low-to-moderate correlations with conceptually relevant tools and by the moderate-strong relationship between the SoNA subscale and obsessive-compulsive (OC) symptoms (*r* = 0.35). We conclude that the SoAS seems to isolate people's general beliefs in their agency from their perceived success in obtaining outcomes.

## Introduction

After being the focus of much psychological research (e.g., Abramson et al., 1978; Newman et al., 1983), the construct of agency and its nomological relatives (such as “freedom,” “control,” “authorship,” “free will,” and “helplessness”) have, since the mid 1980's, largely disappeared from central stage. However, the last few years have seen a resurge of interest in the topic, driven mostly (but not solely) by the neurosciences, which largely focused on the precursors of the sense of agency (e.g., Aarts et al., 2012; David, 2012). The sense of agency (SoA) was defined by Synofzik et al. (2013) as “the registration that I am the initiator of my actions” and studies exploring agency defined in this manner generally use two main methods: “direct” and “indirect” measures (De Houwer, 2003). Direct measures usually include rating scales and self-report questionnaires that explicitly ask about various facets of a person's sense of agency over a specific event. Indirect measures, in contrast, are not direct questions about the extent to which a person feels as the agent of the action or effect in question (Dewey and Knoblich, 2014) but are assumed to be contingent on a prior (unintentional, non-deliberate, seemingly unconscious) computation of one's agency in regard to a specific environmental change. There are two phenomena: sensory attenuation and intentional binding (which we elaborate on below), both of which have been empirically shown to be associated with factors relevant to human agency (e.g., volition) or with predictions derived from a prominent model of the judgment of agency—the so called “comparator model” (Feinberg, 1978; Frith, 1987)^1^.

In contrast to such indirect measures of agency, direct measures may attempt to measure one's sense of agency by asking, for example, *to what degree a person believes he/she was responsible for a perceptual change* (Aarts et al., 2005; Haggard and Chambon, 2012) or *to what degree did he/she feel “in control” while playing a computer game* (e.g., Metcalfe and Greene, 2007) or *to what degree did she think that her action brought about the effect* (see Frith, 2013). Interestingly, although such “local” explicit judgments of agency were used, researchers have consistently found them to be uncorrelated with the implicit measures described above (Kumar and Srinivasan, 2013), experimental manipulations of people's beliefs in their agency within a specific situation were repeatedly documented to affect indirect measures. One such example is the modulation of the “intentional binding” phenomenon (Haggard et al., 2002)—the “compression” in the perceived time interval between performing a voluntary action and observing its effect. In recent years, the intentional binding effect has become a proxy for SoA (Moore et al., 2012; Saito et al., 2015). This phenomenon has also been shown to be sensitive to perceivers' expectancies and beliefs of control over the to-be-bound effect. For example, when varying degrees of automatisation were inserted in the control over an action-effect in the context of an aircraft navigation task (from full operator control to full automatic control)—intentional binding was shown to occur as a function of perceived degree of manual control over the system (Berberian et al., 2012). Similarly, self-reported intentions to produce (or to stop) an effect were also shown to modulate intentional binding (Engbert and Wohlschläger, 2007) as were experimentally manipulated beliefs regarding the source of the effect (self vs. other agent; Desantis et al., 2011; Haering and Kiesel, 2012).

Consciously held beliefs or expectancies about one's SoA were also found to modulate the second indirect measure of SoA—the sensory attenuation effect. Sensory attenuation is the reduction in the perceived intensity of haptic (Blakemore et al., 1998, 1999), auditory (Weiss et al., 2011; Reznik et al., 2015), and visual (Gentsch and Schütz-Bosbach, 2011) stimuli that are perceived as being produced by one's own actions. Experimentally manipulated perceptions of the source of the effect (self vs. other) were shown to modulate the sensory attenuation of effects—with ostensibly self-produced effects being attenuated more than ostensibly other-produced ones (e.g., Desantis et al., 2012).

Thus, although direct measures of “local” or highly contextualized SoA (e.g., “to what degree did you cause the stimulus to flash?”) were often found to be unrelated to indirect measures of SoA in the same contexts (however, see Karsh et al., 2016), a significant amount of empirical evidence shows that experimentally manipulating people's cognitions regarding their own agency does have a causal effect in modulating such implicit measures of agency. In fact, the evidence for the effect of such consciously held beliefs^2^, as well as other lines of work on SoA (e.g., Wegner et al., 2003) have warranted a revision to the dominant model of SoA (Synofzik et al., 2007, 2013; Gentsch and Schütz-Bosbach, 2015). The key revision in the model is the recognition of the role of *judgments of agency* that are argued to stem from one's agency-related cognitions. These are contrasted with *feelings of agency* (one source of influence on judgments of agency) that stem largely from the motor system. Although this revised model has been well accepted by researchers of SoA, surprisingly little empirical work has been conducted to explore the connections between cognition of agency (i.e., SoA-relevant cognitions) and the basic processes assumed to drive SoA, seemingly indexed by the indirect measures. One possible reason for this is the lack of a valid and reliable tool for measuring such *decontextualized, cross situational* (or “chronically held”) cognitions (Eitam and Higgins, 2010).

Our search for existing, potentially relevant tools for directly assessing the sense of agency identified measures that are indeed conceptually related to the general SoA but are either context-specific, such as a recent measure that probes the disruption of SoA during hypnosis (Polito et al., 2013), or measures that assess related concepts but do not directly capture the SoA as defined above, such as Self-efficacy (Bandura, 1977), Locus of Control (Rotter, 1966) or Sense of Control (Lachman and Weaver, 1998). Other existing measures that are seemingly related but are even further removed from the current nature of the study of human agency include those probing for very general and abstract philosophical beliefs, like endorsement of determinism of fatalism (Paulhus and Carey, 2011). The goal of the present project was to develop and test a measure designed to directly assess the general SoA. To this end, we assessed the factor validity of the newly developed scale (Study 1) and subsequently cross-validated the selected factor structure and evaluated the instrument's construct validity (Study 2).

## Study 1–scale design

### Item generation

In the first phase of the SoAS (Sense of Agency Scale) development we aimed to define the item domain, capturing the broad sense of the construct. Based on a review of relevant psychological literature on the sense and judgment of agency (Berti and Pia, 2006; Metcalfe and Greene, 2007; Synofzik et al., 2007; David et al., 2008; Desantis et al., 2011; Haggard and Chambon, 2012; Moore and Obhi, 2012) and following a similar procedure by Polito et al. (2013), we attempted to describe the phenomenological, cognitive, and meta-cognitive experience of agency (or the lack thereof). The item domain is thus constituted by statements describing one's own SoA-relevant experiences. It should be noted that in the case of the SoA construct as described here, such statements do not relate to any *specific* actions or effects, but rather to one's “summary” of her experience of self-agency. Based on the literature referenced above, the subjective experience of agency could be characterized very differently; hence, we attempted to generate items tapping into multiple aspects of the agency experience, such as a controlling self (e.g., “I am in full control of what I do”), a physical self (e.g., “My movements are automatic—my body simply makes them”) or one's interaction with the environment (e.g., “I can't predict how my actions will affect my environment”).

With respect to item wording, per our goal to quantify one's cross situational or “chronic” SoA, we attempted to generate statements reflecting one's context-independent experiences of self-agency, as well as statements corresponding to one's context-independent experiences of lack of agency. The total number of items created was 36, of which 20 were worded to capture lack of agency experiences.

### Item selection

With an initial set of 36 different statements, we proceeded to the next step of refining the item selection based on the assessment of content validity.

First, items were subjected to peer evaluation of content validity by doctoral students and faculty who were not part of this study but are knowledgeable on the topic. Second, the entire item set, originally worded in English, was translated to Hebrew and subsequently back-translated. Following these steps, the item set was administered over the internet to 236 participants drawn from a student participant pool at the University of Haifa.^3^ with responses recorded on a scale from 1 (*strongly disagree*) to 7 (*strongly agree*). The mean age of the entire participant pool population was 24.3 (*SD* = 3.6), and the population consisted of 24.2% males (Sample demographic data were lost due to technical malfunction). Apart from completing the scale, the first 40 respondents were inquired about item intelligibility, ambiguity and clarity.

Subsequently, in an effort to reduce the number of items and refine the instrument while maintaining content validity, 13 items were selected based on the evaluation of content validity, item response variability, pilot study participants' ratings of item clarity and the magnitudes inter-item correlations; while attempting to keep at least a roughly equal number of “agency experience” and “lack of agency experience” items (6 and 7, respectively).

### Exploratory factor analysis

An exploratory factor analysis was performed on the 13 items in CEFA (Browne et al., 1998) using GLS as the discrepancy function (the first three eigenvalues were 4.3, 1.7, and 1). Originally, we expected a unidimensional solution; however, although a one-factor model yielded a satisfactory fit, $\chi_{\left(65\right)}^{2}$ = 124.8, RMSEA = 0.063 (90% CI = 0.046, 0.079), RMSP = 0.11, the matrix of residuals contained meaningful clusters of unexplained item covariance. Thus, two- and three-factor models using the same discrepancy function and an oblique Quartimax rotation were also investigated. We concluded that the two-factor model performs optimally, $\chi_{\left(53\right)}^{2}$ = 70.5, RMSEA = 0.037 (90% CI = 0.000, 0.059), RMSP = 0.05, while keeping the model relatively simple and the factors most easily interpretable. The three-factor model, $\chi_{\left(42\right)}^{2}$ = 47.8, RMSEA = 0.024 (90% CI = 0.000, 0.059), RMSP = 0.04, resulted in only a slight additional decrease in the magnitude of the residual matrix elements at the expense of adding an additional latent variable of problematic interpretability. Table 1 shows the two-factor model loadings after rotation. The two factors were moderately correlated (*r* = −0.39).

**Table 1:** Factor loadings of items after rotation.

	**Factor**	
**Item**	**SoPA**	**SoNA**
1. I am in full control of what I do	**0.66**	−0.07
2. I am just an instrument in the hands of somebody or something else	−0.22	**0.44**
3. My actions just happen without my intention	0.01	**0.71**
4. I am the author of my actions	**0.44**	−0.39
5. The consequences of my actions feel like they don't logically follow my actions	−0.26	**0.38**
6. My movements are automatic—my body simply makes them	0.17	**0.69**
7. The outcomes of my actions generally surprise me	0.01	**0.56**
8. Things I do are subject only to my free will	**0.80**	0.12
9. The decision whether and when to act is within my hands	**0.53**	−0.25
10. Nothing I do is actually voluntary	−0.09	**0.57**
11. While I am in action, I feel like I am a remote controlled robot	−0.11	**0.52**
12. My behavior is planned by me from the very beginning to the very end	**0.63**	0.02
13. I am completely responsible for everything that results from my actions	**0.51**	−0.04

*SoPA, Sense of Positive Agency, SoNA, Sense of Negative Agency. Generalized Least Squares with Quartimax rotation. The higher of the two loadings appears in bold*.

Given item content, we labeled the first factor “Sense of Positive Agency (SoPA)” and the second factor “Sense of Negative Agency (SoNA).” For the interpretation of the factors, as well as for an argument on whether the two should be understood as distinct constructs or, rather, as two facets of the same construct, see the section General Discussion.

### Reliability

The reliability of the two subscales was (McDonald's) ω = 0.78 (95% CI = 0.73, 0.82) and ω = 0.76 (95% CI = 0.71, 0.81) for the SoPA and SoNA, respectively (confidence intervals created using bias-corrected and accelerated bootstrapping with 1,000 replications). To obtain an estimate of the constructs' stability over time, the original sample was contacted again 2 months after the first wave of data collection and the scale was administered for the second time. Ninety-one participants provided their answers, and test-retest reliabilities (calculated as latent correlations) were *r* = 0.78 for the SoPA and *r* = 0.74 for the SoNA, supporting the interpretation that we are indeed estimating people's cross-situational SoA.

## Study 2–confirmatory factor analysis and validity assessment

### Confirmatory factor analysis

**Figure 1:**
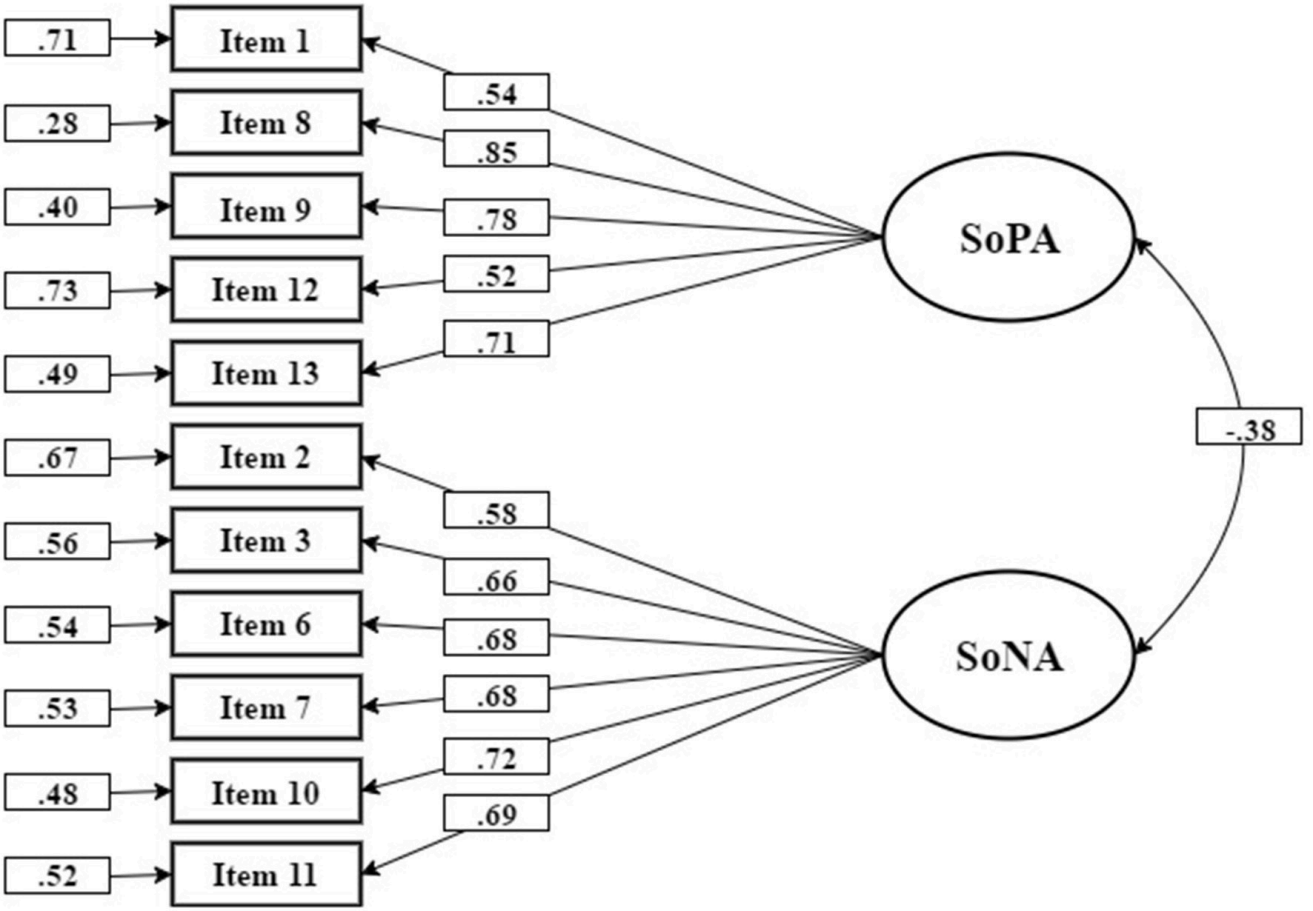
The structure and parameters of the two-factor confirmatory model. SoPA, Sense of Positive Agency, SoNA, Sense of Negative Agency. The figure presents (from left to right) standardized residuals, standardized loadings and the correlation between the two latent variables. Variances of both latent variables were constrained to 1 for identification purposes.

In the next phase, data from a community sample of 408 participants (mean age of 39.8, *SD* = 16.1, 51% males) were collected using a commercial on-line panel in Israel (Midgam, http://www.midgampanel.com). The two-factor model from Study 1 was fit to the data using GLS estimation in lavaan (Rosseel, 2012); two additional items (item 4: I am the author of my actions; and item 5: The consequences of my actions feel like they don't logically follow my actions) were removed before the analysis was performed since they substantially cross-loaded on both factors in the preceding exploratory analysis. The variances of both latent variables were fixed at 1 for model identification. The model fit was satisfactory, $\chi_{\left(43\right)}^{2}$ = 93.0, RMSEA = 0.054 (90% CI = 0.039, 0.069), RMSP = 0.06, CFI = 0.83; the correlation between factors was *r* = −0.38, which is practically identical to the correlation estimated in Study 1. Note that incremental fit indices such as CFI are not very informative here since the baseline RMSEA is 0.116 (less than 0.158; Kenny et al., 2015). Figure 1 contains the model diagram with parameter estimates.

### Reliability

Reliability estimates were again computed using the new data, with reliability estimated at (McDonald's) ω = 0.80 (95% CI = 0.76, 0.83) and ω = 0.75 (95% CI = 0.67, 0.79) for the SoPA and SoNA, respectively (confidence intervals created using bias-corrected and accelerated bootstrapping with 1,000 replications).

### Assessment of validity

Data on multiple relevant constructs were also collected in order to assess the construct validity of the SoAS. For each construct, the measurement instrument^4^ and empirical results are described and discussed below. Table 2 shows the latent correlations between these constructs and the two SoAS factors, obtained from a structural model fit in lavaan (Rosseel, 2012). The structural model allowed for correlations between all latent variables to be estimated, and was fit using a robust DWLS discrepancy function. The structural model fit the data well, $\chi_{\left(5780\right)}^{2}$ = 8246.81, RMSEA = 0.037 (90% CI = 0.035, 0.038), CFI = 0.89 (note again that the baseline RMSEA was 0.108; and so incremental fit indices like CFI are less informative; Kenny et al., 2015).

**Table 2:** Latent correlations between the SoAS factors and other constructs from Study 2.

	**SoPA**	**SoNA**
GSES–Initiative	−0.27^*^	0.35^*^
GSES–Effort	0.43^*^	−0.31^*^
GSES–Persistence	−0.31^*^	0.34^*^
PSE–Ability	0.16^*^	−0.06
PSE–Confidence	0.45^*^	−0.45^*^
External Locus of Control	−0.35^*^	0.33^*^
FAD-Plus–Free Will	0.49^*^	−0.26^*^
FAD-Plus–Scientific D	0.15	0.10
FAD-Plus–Fatalistic D	−0.07	0.35^*^
FAD-Plus–Unpredictability	0.12	0.24^*^
BC–Private	0.02	0.08
BC–Public	0.09	0.06
BC–Body Competence	0.24^*^	−0.10

*SoAS, Sense of Agency Scale; SoPA, Sense of Positive Agency; SoNA, Sense of Negative Agency; GSES, General Self-Efficacy Scale; Scientific D, Scientific Determinism; Fatalistic D, Fatalistic Determinism; PSE, Physical Self-Efficacy; BC, Body Consciousness*.

* *Significant at p < 0.05*.

### General self-efficacy

While Self-efficacy theory and corresponding measures emphasize the importance of domain-specificity (Bandura, 1977; see below results obtained using a domain-specific Self-efficacy measure), generalized beliefs in Self-efficacy have been shown to be a useful predictor of “ego strength” and beliefs in personal control (Sherer et al., 1982). General Self-efficacy (GSE) represents a generalized, positive belief in personal competence and ability to organize and execute desired (i.e., goal-directed) behavior. As SoA is often confounded by the one's sense of being effective in attaining one's desired outcomes (Higgins, 2011), this construct is highly relevant to the SoA (or the lack of it). The question is to what degree the SoAS differentiates between beliefs about one's general effectiveness in attaining goals and one's sense of agency. In order to empirically evaluate the degree of similarity between the SoPA/SoNA constructs and GSE, we administered the General Self-efficacy Scale (GSES, Bosscher and Smit, 1998) which reflects three underlying aspects of GSE—the willingness to initiate behavior (Initiative; note however, that the “Initiative” items in GSES, actually measures the lack of initiative), the willingness to expend effort to continue behavior (Effort) and the willingness to persevere in behavior despite hardship (Persistence; again, the wording of items in the GSES implies the opposite of persistence). As can be seen in Table 2, the size of the correlations indicates that a substantial amount of the SoAS variance was unexplained by the GSE, supporting the conclusion that the GSE and the SoA as measured by the SoAS measure different, albeit related, constructs.

### Physical self-efficacy

For the sake of conceptual consistency, we also examined the relationship between the SoA as measured by the SoAS and an agency-relevant *domain-specific* form of Self-efficacy (Bandura, 1977). We administered the Physical Self-efficacy Scale (PSE; Ryckman et al., 1982), measuring beliefs in personal competence specifically related to one's physical body, and further distinguishing between perceived physical ability and self-presentation confidence. We selected *Physical* Self-efficacy because proximal control over our own body is arguably the “core” of agency beliefs (Elsner and Aschersleben, 2003). We found that the Self-Presentation Confidence PSE subscale (but not the Ability subscale) exhibits a moderately-strong relationship with both SoAS subscales (in the predicted direction). This finding suggests that being pleased with one's physique is related to one's sense of control of the body, mind and the environment. We speculate on the nature and directionality of this relationship in the section General Discussion.

### Locus of control

The findings regarding self-efficacy suggest that SoA relevant beliefs—as measured by the SoAS—are not merely specific or general beliefs in personal competence, whether physical or otherwise. However, it is possible that the SoAS items may reflect a close conceptual relative of Self-efficacy—the Locus of Control (LOC; Rotter, 1966), that is, individuals' beliefs regarding their control *over obtaining desired outcomes*. Note that in some previous experimental and theoretical work authors differentiated both conceptually and operationally between motivation stemming from working toward and obtaining desired outcomes and motivation which stems from working toward and obtaining control (Eitam et al., 2013; Karsh and Eitam, 2015; Karsh et al., 2016 for conceptual analyses see White, 1959; Higgins, 2011). To address the association between these two motivations when measured by self-reports, we administered Rotter's (1966) LOC scale. The correlations between LOC and the two SoAS factors were fairly modest, lending support to the conceptual difference between judgments of having control over obtaining desired outcomes (or of not having undesired ones) and the SoA.

### Free will and determinism beliefs

As we have seen so far, the SoAS seems to measure a unique construct, differing empirically from beliefs in personal competence and Locus of Control. However, another viable possibility is that the pattern obtained above stems from that fact that the SoAS, rather than measuring people's beliefs about their *own* agency (i.e., *their* SoA) simply reflects lay, culturally transmitted perceptions of the philosophical notions of free will, unpredictability and/or determinism. Although the SoAS items do not directly probe for such lay theories, they might indirectly capture them nonetheless. To examine this possibility, we administered the Free-Will and Determinism Beliefs Scale (FAD-Plus; Paulhus and Carey, 2011). The FAD-Plus consists of four distinct subscales which target beliefs in 1: Free Will, 2: Fatalistic Determinism, 3: Scientific Determinism, and 4: (ontological) Unpredictability. As seen in Table 2, the four FAD-Plus subscales were differently related to the SoAS subscales—specifically, beliefs in free will were moderately related to the SoPA subscale and their relationship to the SoNA subscale was substantially weaker. This pattern suggests that SoPA is more related to philosophical concepts of personal autonomy and responsibility than SoNA. The opposite can be said about Fatalistic Determinism, which is not at all related to SoPA but was associated with SoNA, suggesting the latter's connection to assumptions on the role of fate and unchangeable destiny. The pattern of correlations also lends support to the conceptual differentiation between the SoAS factors and further indicates that SoNA, rather than merely reflecting the *lack* of a sense of agency, might reflect something akin to the endorsement of “existential helplessness.” Additional support for the conclusion that the SoNA subscale reflects such helplessness is that it was weakly and positively related to Unpredictability (while the SoPA subscale was not). Interestingly, Scientific Determinism, which most clearly represents the endorsement of the philosophical stance that biological and environmental forces dominate human behavior and personality, did not correlate with either subscale.

### Body consciousness

Up to this point we have presented findings that both differentiate between the SoA as measured by the SoAS from other closely related beliefs about the self and relevant lay theories and show its relationship to them. In addition, we evaluated the degree to which the SoAS taps into certain aspects of self-monitoring and bodily awareness that have been recently linked to the so-called “minimal self” (Aspell et al., 2013; for a recent review see Blanke, 2012). At least in relation to phenomenal experiences, scholars have differentiated between *body ownership* (the feeling that this body/body part is mine) and SoA. This was particularly relevant as SoAS includes items that directly refer to one's body such as “*My movements are automatic—my body simply makes them”* or “*While moving and acting, it feels like I am a remotely controlled robot”* which may capture individual differences in body monitoring and hence may be related more strongly to body ownership than to the SoA. In order to quantify the relationship between one's attention to the body, its states and the SoAS scores, we administered the Body Consciousness Questionnaire (BC, Miller et al., 1981), which measures three distinct aspects—Private Body Consciousness, Public Body Consciousness and Body Competence, the latter being the only strictly evaluative aspect of bodily awareness. Based on the magnitude of correlations, neither aspect seems to be substantially related to the SoAS, indicating that our new scale does not capture bodily monitoring or variation in one's feeling of body ownership.

### Interim conclusion

The relationships of conceptually relevant constructs to the two SoAS factors, one capturing cognitions of *positive agency* and the other cognitions of *negative agency* (tentatively described as being akin to “existential helplessness”). While these two subscales coherently relate to conceptual neighbors such as Locus of Control and beliefs in free will, they are sufficiently dissimilar to warrant their unique measurement. For example, negative agency seems not to be a “scientific” or “cold” form of determinism, but rather a fatalistic, pessimistic, and potentially de-motivating kind. This is intriguing, considering that the SoAS does not measure whether an individual feels that he/she perpetually *fails to meet her goals*—which would be associated with anhedonia or hopelessness—rather, the SoAS focuses on control of rudimentary, basic (maybe even mundane) faculties, such as locomotion or the stream of thought.

### Incremental validity

Beyond establishing that while the SoA as measured by the SoAS relates to relevant constructs, it is not redundant with any one of them we wanted to test its validity by examining whether it has any incremental value over and above the instruments and/or constructs reviewed above. Given that recent work has focused on the SoA of people suffering from obsessive-compulsive (OC) tendencies and disorder (e.g., Rossi et al., 2005; Belayachi and Van der Linden, 2010; Gentsch et al., 2012; Oren et al., 2016), we chose to examine whether our measure, which appears to capture unique elements of SoA, would correlate with the severity of OC symptoms beyond the measures presented above (i.e., its conceptual relatives).

The phenomenology of compulsions, one of the defining features of OCD, implies a deficient SoA, as individuals with OCD chronically experience that they do not choose some of their actions freely but are compelled to act in certain ways. More generally, a central assumption in Shapiro's (1965) classic theory of OCD is that obsessive-compulsive (OC) individuals have a deficient sense of autonomy and agency. Nevertheless, only a handful of studies have empirically examined the SoA in OCD patients, and fewer yet have examined basic processes believed to contribute to the SoA. For example, one study (Gentsch et al., 2012; see also Rossi et al., 2005) examined EEG responses of OCD and control participants to self-generated vs. externally generated visual stimuli and found the that the suppression of the N1 component was reduced in OCD participants (seemingly related to muted sensory attenuation—see section Introduction).

While these observations indicate a diminished SoA in OCD patients, other findings appear to lead to the opposite conclusion. The OCD-related construct of inflated responsibility (Salkovskis et al., 1999), for instance, suggests a heightened SoA in this population. Similarly, OCD patients often believe that their thoughts would automatically lead to actions (“thought-action fusion”; Shafran et al., 1996) or events in the world (“thought-event fusion”; Gwilliam et al., 2004), which also appears to suggest an elevated SoA. In line with these observations, Reuven-Magril et al. (2008) found an increased illusion of control in participants with high OC tendencies as well as in OCD participants. A similar duality was observed in the study by Gentsch et al. (2012): while the EEG indices in OCD participants suggested less agency as indexed by sensory attenuation, the direct (contextualized) probes of agency were higher in OCD participants compared to controls when participants rated the relation between their actions and visual stimuli. Furthermore, these direct judgments of agency were correlated with the severity of OC symptoms.

In sum, both the phenomenology of OCD and extant research findings suggest a distortion in the SoA in OCD, but the direction of the distortion appears to vary. We reasoned that by measuring one's cross situational or “chronic” sense of agency with the SoAS, we would be able to measure core beliefs about own agency in OC individuals. Furthermore, both Self-Efficacy and Locus of Control were shown to be related to both OC tendencies and depression (e.g., Molinari and Niederehe, 1985; Ehrenberg et al., 1991; Scholz et al., 2002). We therefore administered two widely used and psychometrically sound instruments—the Beck Depression Inventory-II (BDI-II, Beck et al., 1996) and the Obsessive-Compulsive Inventory Revised (OCI-R; Foa et al., 1998). The question we posed here was whether either of the SoAS subscales would explain any significant amount of variance in OCI-R scores that is not explained by Self-Efficacy or Locus of Control alone. Given the substantial correlation between depressive and OC symptomology, we controlled for depressive symptoms using the BDI-II score.

Table 3 contains the latent correlations between each of the SoAS subscales, BDI-II and OCI-R, controlling for the effect of the other candidate predictors (GSES, PSE, and LOC). As can be seen, the SoPA subscale did not correlate with either depressive or OC symptoms, while the SoNA subscale positively correlated only with OC symptoms. That is, negative agency alone was moderately related to OC symptoms, even after depressive symptoms were controlled for. Beyond the evidence for incremental validity of the SoAS instrument, we see this as a theoretically important finding that we develop further in the section General Discussion.

**Table 3:** Correlations of SoPA and SoNA with BDI-II and OCI-R after controlling for related constructs.

	**SoPA**	**SoNA**
BDI-II	−0.01	0.03
OCI-R	−0.11	0.35^*^

*For the correlations with BDI-II above, the effects of GSES, PSE, LOC, and OCI-R are controlled. For the correlations with OCI-R above, the effects of GSES, PSE, LOC, and BDI-II are controlled. BDI-II, Beck Depression Inventory-II; OCI-R, Obsessive-Compulsive Inventory Revised; GSES, General Self-Efficacy Scale (all three factors); PSE, Physical Self Efficacy (both factors); LOC, Locus of Control*.

* *Significant at p < 0.05*.

## General discussion

We presented empirical evidence supporting the validity of the SoAS as a direct measure of cross-situational or “chronic” sense of agency. In what follows, we briefly highlight a few empirically driven and conceptual points that emerged during the process of developing and evaluating the SoAS.

### Dissociating sense of agency from effectiveness in obtaining desired outcomes

The Sense of Agency Scale (SoAS) was developed as a tool for measuring individuals' beliefs about being agents in the sense of generally experiencing control over one's body, thought and immediate environment. Such a tool can enable the quantification and dissociation between (1) the influences of experiencing success in having what one needs and/or desires and (2) experiencing success in controlling the environment and/or oneself (White, 1959; Eitam and Higgins, 2010; Higgins, 2011; Eitam et al., 2013; Karsh and Eitam, 2015; Karsh et al., 2016). Indeed, this dissociation was nicely demonstrated by the pattern of correlations obtained between the SoAS and the two measures of Self-Efficacy (both general and specific). Self-Efficacy, reflects one's agency in the sense of being “free” to perform an action that is key to goal attainment (e.g., approaching a Boa constrictor to reduce a debilitating snake phobia; Bandura, 1977). Conversely, the SoAS is designed to measure SoA dissociated from instrumentality or goal-relevance.

### Direct and indirect measures of the sense of agency

As stated in the section Introduction, recent years have seen a resurgence of empirical interest in the sense of agency, whether by directly measuring peoples' conscious-deliberate judgements of the current degree of control they have over an experimental situation or of “authoring” (i.e., generating) a specific experimentally induced perceptual outcome or via indirectly indexing it through a number of phenomena such as intentional binding. Surprisingly, recent work has shown that the two types of measures can be uncorrelated (Kumar and Srinivasan, 2013; Dewey and Knoblich, 2014) and/or that the correlation between them may depend on the salience of factors influencing them and on the specific measures used (Nisbett and Wilson, 1977; Karsh et al., 2016).

Previous studies using direct measures of SoA have asked participants directly about their SoA in regard to specific actions or effects that occurred in the context of the experiments they participated in (e.g., Reuven-Magril et al., 2008; Gentsch et al., 2012). The SoAS measure, in contrast, is a direct measure that targets one's “chronic” or cross-situational experience of agency, as distinct from how it unfolds in a specific experimental task or for a specific experience of agency. This was done by asking participants about their general perceptions and cognitions regarding their own SoA in general and in regard to multiple aspects of the experience of agency. As such, the SoAS may enable some structuring of the complicated and often-conflicting pattern of findings obtained by direct and indirect measures of SoA and of the relationships among them by enabling consistent measurement of individual differences in (or situational effects on) “global” sensing of agency.

### One construct with two facets or two constructs?

The first major finding generated by the SoAS is that what we have termed “Sense of Positive Agency” (SoPA)—essentially feeling in control of one's body, mind and environment—is only moderately correlated with the “Sense of Negative Agency” (SoNA)—the feeling that the above are *not* under one's control^5^. We made sense of the two factors and their estimated correlation in line with the neuroscientific evidence of anatomical differentiation between “positive” (“I am the agent”) and “negative” (“I am not the agent”) agency judgments (Farrer and Frith, 2002; Farrer and Franck, 2003; Nahab et al., 2011; Sperduti et al., 2011). Further favoring the differentiation between “positive” and “negative” SoA is the intriguing evidence collected from epileptic patients whose brains were directly stimulated at the anterior cingulate cortex region (ACC; argued to be involved in motor and cognitive control, among other things) and who consistently, during stimulation, reported a feeling of a looming “ominous event” which they cannot control or handle (Parvizi et al., 2013). The “negative” agency may be a *generalized* case of the one captured by the literature which focused on learned helplessness (for reviews see Maier and Seligman, 1976; Abramson et al., 1978), which is the special case in which the (usually aversive) external environment is not under one's control—a case that is cross-situational (or “chronic”) and cross-domain rather than focusing on a specific instance or aspect of the experience of agency. At this stage we can only speculate at what might be the impact of scoring high on Negative Agency but given the passivity generated by the far more local “learned helplessness” we are assuming it may have severe consequences regarding people's motivation to act (see also section Sense of agency and psychopathology).

Further support for the differentiation between positive and negative agency as well as for the hypothesized relationship between negative agency and helplessness comes from a recent study (Karsh et al., unpublished data). In this study the participants' degree of control over noxious electrical current applied to the participant's finger (either controlled by the participant or applied automatically by a controlling computer) and its temporal predictability (timing fully predictable or not) were manipulated. Although the authors expected to find a strong negative correlation between self-reported feelings of being in control and feeling helpless, the two were only weakly correlated (Pearson's *r* = 0.3). Thus, it is possible that two SoAS subscales, SoPA and SoNA, map onto the feeling of control and of a variant of helplessness, correspondingly. Interestingly, as stated above, different brain regions were shown to be involved in situations in which participants had an objective or subjective experience of control compared to ones in which they had no control over mundane effects. Thus, although more research is needed, we propose that, at least when one's “global” sense of agency is concerned, the seemingly continuous sense of agency could be parsed into two different judgments—*having control* and being *existentially helpless* or, alternatively, to the “hot” affective-counterpart of not having control (Karsh et al., unpublished data).

### Sense of agency and psychopathology

A second finding generated by the SoAS is the surprisingly high correlation between the SoNA factor and the degree of reported obsessive-compulsive (OC) symptoms, even after controlling for the relationship between OC symptoms with depression as well as for a number of close concepts (including the SoPA subscale). On one hand, this finding is commonsensical—the more people suffer from disrupting, intrusive thoughts and the more they fail in their attempts to control these thoughts, the less control they would likely feel. Still, the literature on the relationship between OCD symptomology and the sense of control doesn't paint such a clear picture. In fact, one could have made the opposite prediction in that people who perform acts with the belief that these will prevent looming disastrous events (e.g., a loved one's death) could entertain having omnipotent control. Along the same lines, Pacherie (2008) hypothesized that the characteristic feature of OCD is an abnormally low SoA that may be counteracted by rituals; these serve to create an illusory SoA that then helps to reinstate the desired feeling of control (see also Reuven-Magril et al., 2008). In other words, it may well be that a highly OC person experiences fluctuations in self-agency and sense of control. This may motivate compensatory efforts to control all actions, thoughts, impulses and emotions. Such a compensatory mechanism is exhibited when a person with OCD tries to prevent negative events, on which he/she has no control, by controlling what he/she does, thinks, desires, or feels. It is possible that by distilling the core of the agency experience (most importantly from the contribution of success or failure in attaining positive outcomes to one's sense of agency), the SoAS has managed to capture the magnitude of uncontrollability/helplessness that OC symptoms generate.

The fact that our findings are consistent with those obtained with indirect measures (Rossi et al., 2005; Gentsch et al., 2012; Oren et al., 2016) suggests that the assessment of SoA through direct measures can capture the more primary, diminished SoA in high OC individuals. What may help explain the existing discrepancy between indirect and direct measures is that in previous studies, direct probes of agency measured the SoA derived from a specific action conducted by the participants (e.g., Reuven-Magril et al., 2008; Gentsch et al., 2012) while we asked participants about their general cognitions and perceptions regarding their SoA. Future studies could further examine the degree to which extant indirect measures of SoA correlate with the SoAS.

### Future directions

Our goal was to develop a measure of “chronic” or general belief in having “core” agency. One of the purposes for creating this measure was to enable the investigation of whether such general beliefs would modulate indirect judgments of agency—in other words, whether they would be related to (the currently used) indirect measures of agency, such as intentional binding (Haggard et al., 2002) or sensory attenuation (Desantis et al., 2012). Therefore, employing both SoAS and implicit measures of agency in the same study is the next necessary step in this line of research.

It should be noted that the results presented here should be viewed as preliminary and as a first step toward attempting to measure “chronic” sense of agency. Another caveat is that our data were collected using the Hebrew version of the SoAS and thus the conclusions presented cannot be automatically applied to other languages. A validity study of an English translation is warranted before the SoAS can be sensibly used in English-speaking samples.

Finally, the intriguing and substantial correlation between OC symptoms and negative agency definitely warrants systematic exploration. A reasonable first step would be to see whether the pattern found holds in OCD-diagnosed patients and to continue testing whether indirect measures of agency also follow this pattern. If they do, this could address the apparent discrepancy in regard to SoA of people suffering from OCD reviewed above. A second step may be an experience sampling study with people diagnosed with OCD using a modified version of the SoAS targeting daily (or even hourly) fluctuations in the SoA. Such monitoring augmented by collecting information on people's internal and external experiences would allow the identification of key factors driving the negative sense of agency. In turn, the identification of such factors may enable zeroing in on the malfunctioning processes underlying this (agency bound) disorder.

## Author contributions

AT and BE: Acquisition, Analysis and interpretation of the data, design of the study, drafting the work and revising it critically, final approval. EO and RD: Interpretation of the data, design of the study, drafting the work and revising it critically, final approval.

### Conflict of interest statement

The authors declare that the research was conducted in the absence of any commercial or financial relationships that could be construed as a potential conflict of interest.

## Footnotes

^1^Another phenomenon—“motivation from control”—has also been recently demonstrated to be sensitive to very similar factors and hence may be a third indirect index of SoA (Eitam et al., 2013; Karsh and Eitam, 2015; Karsh et al., 2016).

^2^As often is the case in the debate regarding the *causal* role of *consciously* held beliefs it is impossible to rule out the possibility that the agency-relevant information given to participants (e.g., the appearance of the “X” symbol will indicate that in the coming trial you will have no control) affects the basic (seemingly unconscious) processes involved in so-called implicit judgment of agency *directly*, rather than affecting them by being *consciously* held.

^3^This study was carried out in accordance with the recommendations of The Institutional Review Board (IRB) at the University of Haifa. with written informed consent from all subjects. All subjects gave written informed consent in accordance with the Declaration of Helsinki. The protocol was approved by The Institutional Review Board (IRB) at the University of Haifa..

^4^Hebrew versions of the instruments described here were used in the study.

^5^Note that it is not the case that the items of the SoNA subscale are worded negatively.
